# Structure Inhomogeneity of Gold Nanoparticles and Its Effect on H_2_ Dissociative Chemisorption

**DOI:** 10.3390/nano16100570

**Published:** 2026-05-07

**Authors:** Andrey K. Gatin, Sergey Yu. Sarvadii, Polina K. Ignat’eva, Ekaterina I. Rudenko, Maxim V. Grishin, Dinara Tastaibek, Denis A. Yavsin, Sergey A. Gurevich

**Affiliations:** 1 N.N. Semenov Federal Research Center for Chemical Physics of Russian Academy of Sciences (FRCCP RAS), Kosygina Street 4, 119991 Moscow, Russia; akgatin@yandex.ru (A.K.G.); polina_ignateva_2002@bk.ru (P.K.I.); ekaterina.rudenko@chph.ras.ru (E.I.R.); mvgrishin68@yandex.ru (M.V.G.); 2 Department of Materials Science, Nanotechnology and Engineering Physics, Mining and Metallurgical Institute, Satbayev University (KazNRTU), Satbaeva Street 22, 050013 Almaty, Kazakhstan; baimukhambetovadinara@gmail.com; 3 Ioffe Physico-Technical Institute, Russian Academy of Sciences, Politechnicheskaya Street 26, 194021 Saint Petersburg, Russia; yavsin@mail.ioffe.ru (D.A.Y.); gurevich@quantel.ioffe.ru (S.A.G.)

**Keywords:** nanoparticles, gold, size effect, amorphous structure, surface electronic states, hydrogen chemisorption, scanning tunneling microscopy, scanning tunneling spectroscopy

## Abstract

Significant differences in hydrogen adsorption on amorphous and crystalline gold nanoparticles deposited on highly oriented pyrolytic graphite (HOPG) were revealed. Crystalline nanoparticles were synthesized via the impregnation–precipitation method followed by annealing at 700 K, whereas amorphous ones were obtained using the laser electrodispersion method. The morphology and electronic structure of single nanoparticles were investigated with high spatial resolution using scanning tunneling microscopy and spectroscopy (STM/STS) in ultra-high vacuum both before and after exposure to molecular hydrogen at doses of 400–6000 L. Experiments performed at room temperature showed that the surface coverage by the adsorbate in both cases begins at the Au-HOPG interface, spreads towards the center of the particle, and corresponds to the island growth model. However, amorphous nanoparticles have fewer growth sites at the periphery compared to crystalline ones. The local electronic structure of amorphous nanoparticles is more inhomogeneous compared to crystalline ones, demonstrating variation across different points on the nanoparticle surface. It was shown that dissociative chemisorption of hydrogen takes place on amorphous gold nanoparticles with a size of 4–6 nm. Chemisorption is completely inhibited when the nanoparticle size is reduced to 2 nm or less.

## 1. Introduction

The sensitivity of surface-based processes in metal nanoparticles to their electronic and atomic structure offers a wide range of opportunities to tune their properties [1,2,3]. In nanostructured systems, spatial confinement of electron motion leads to modifications in the electronic state spectrum, giving rise to the ‘size effect’—a complex set of interconnected phenomena, including atomic structure rearrangement [4], lowering of the immiscibility threshold for nanoalloy components [5], alteration of the relaxation time of electronic and structural excitations [6,7], etc. Although the size, shape, and atomic structure of deposited nanoparticles can evolve during chemical reactions, these characteristics are typically established during the synthesis stage and are strongly dependent on the substrate.

Gold demonstrates a striking example of size effects. The influence of nanoparticle size and structure on the activity of hydrogen adsorption and dissociation centers on the gold surface is critically important, for example, for the development of hydrogen energy and fine organic synthesis, where hydrogen activation is a crucial stage in the purification of hydrogen fuel in fuel cells [8] and the hydrogenation of unsaturated compounds [9]. Atomic hydrogen, formed by the dissociation of H_2_, acts as a key intermediate in this process. In this regard, the mechanisms of H_2_ molecule dissociation on gold nanoparticles are a pressing issue. An important aspect is the difference in activation energies for this process on different surface sites of nanoparticles. Various structural elements of the nanoparticle surface—flat facets, edges, and coordinatively unsaturated corners—exhibit different propensities for H_2_ dissociation, which is reflected in varying mechanisms and activation energies [10,11,12]. Thus, the nanoparticle perimeter and the interface with the support play a determining role, and the size and morphology of Au directly influence the distribution of active centers and, consequently, the catalytic activity in hydrogen-involved reactions.

In our previous studies, we investigated the adsorption of oxygen and hydrogen on gold, nickel, and platinum nanoparticles deposited on highly oriented pyrolytic graphite (HOPG) [13,14,15,16,17,18]. The stability of Au–H, Ni–O, and Pt–O adsorption complexes in these nanostructured systems was successfully described using the resonant chemisorption model [13]. The chemical activity of surface sites on the deposited nanoparticles is primarily governed by two factors: the electric charge of the particle and the deformation of its atomic structure due to interaction with the substrate [13]. These studies account for the qualitative trends observed across all three systems, specifically the center-to-periphery spatial distribution of adsorption complexes on the surface of single nanoparticles. In particular, hydrogen chemisorption on gold occurs most actively near the Au–HOPG interface [13,14]. This finding was supported further by quantum chemical simulation, which identifies the nanoparticle periphery as the preferred region for hydrogen adsorption [14].

The formation of Au–H on the surface of gold nanoparticles was demonstrated to be a purely surface-based process, occurring without hydrogen penetration into the bulk [15]. Furthermore, we showed that the Au–H chemical bond formation is primarily driven by the surface electronic states of the gold nanoparticle [14,16]. In the case of gold, this contribution is most pronounced compared to platinum or nickel, owing to the deep position of the *d*-band. This implies that both factors—charging and deformation—must be examined in terms of their influence on the energy and occupancy of surface electronic states.

The effect of nanoparticle charging on Au–H bond formation and its inhibition was examined in [17]. Also some preliminary experiments on water synthesis on amorphous gold nanoparticles were conducted [18]. However, the relationship between atomic structure and surface electronic states remains unclear. Investigating this issue by simply changing the substrate is not feasible, as it would simultaneously affect nanoparticle charging. Therefore, the answer must be sought by controlling the degree of nanoparticle lattice deformation during the synthesis stage. We address this problem in the present paper. Our goal is to establish the influence of structural inhomogeneity of gold nanoparticles—their amorphous or crystalline state—on their ability for dissociative hydrogen chemisorption. For this purpose, we synthesized nanoparticles of two types on HOPG. Crystalline particles of 4–6 nm size were synthesized via the impregnation–precipitation method followed by annealing at 700 K, whereas amorphous nanoparticles were obtained using the laser electrodispersion (LED) method. The morphology and electronic structure of single nanoparticles were investigated with high spatial resolution using scanning tunneling microscopy and spectroscopy (STM/STS) in ultra-high vacuum. Measurements were performed both before and after exposure to molecular hydrogen at doses up to 6000 L. This approach allowed for a comparative analysis of the local density of electronic states, revealed the patterns of hydride phase growth on the surface of nanoparticles with different crystallinity, and enabled the evaluation of threshold sizes at which hydrogen chemisorption becomes possible or, conversely, is completely inhibited.

## 2. Experiment

### 2.1. Nanoparticles Synthesis: Impregnation–Precipitation Method

One of the methods used in this study for gold nanoparticles synthesis was the impregnation–precipitation technique. Plates of highly oriented pyrolytic graphite (HOPG) were used as the substrate, with a *c*-axis deviation from the normal to the basal plane not exceeding 0.8° (as specified by the supplier, AIST-NT, Moscow, Russia). An aqueous solution of H[AuCl_4_]·4H_2_O (AURAT, Moscow, Russia) with a gold concentration of 24 mg/L was used as the precursor for gold nanoparticles. The synthesis was performed as follows: the precursor was applied to the surface of the HOPG plate, after which the sample was dried and transferred into an ultra-high vacuum chamber. Under ultra-high vacuum conditions, the sample was heated to *T* = 700 K for 8 h. It is known that heating leads to the decomposition of HAuCl_4_ via the following reactions [19]:

(1) $$2H[{\mathrm{AuCl}}_{4}]\cdot 4H_{2}O\;\overset{390\;K}{\to }{\mathrm{Au}}_{2}{\mathrm{Cl}}_{6}+2H\mathrm{Cl}+8H_{2}O$$

(2) $$H[{\mathrm{AuCl}}_{4}]\cdot 4H_{2}O\;\overset{430-480\;K}{\to }\mathrm{Au}\mathrm{Cl}+H\mathrm{Cl}+{\mathrm{Cl}}_{2}+4H_{2}O$$

In turn, the formed gold compounds decompose upon heating [19]:

(3) $${\mathrm{Au}}_{2}{\mathrm{Cl}}_{6}\;\overset{450-530\;K}{\to }2\mathrm{Au}\mathrm{Cl}+2{\mathrm{Cl}}_{2}$$

(4) $${\mathrm{Au}}_{2}{\mathrm{Cl}}_{6}\;\overset{560\;K}{\to }2\mathrm{Au}+3{\mathrm{Cl}}_{2}$$

(5) $$2\mathrm{AuCl}\;\overset{560\;\;K}{\to }2\mathrm{Au}+{\mathrm{Cl}}_{2}$$

Therefore, the selected annealing temperature of 700 K ensures the formation of metallic gold on the HOPG surface.

Studies have shown that the impregnation–precipitation method leads to the formation of metal nanoparticles with an atomic structure close to that of a single crystal [20,21]. Hereinafter, the term ‘crystalline nanoparticles’ will refer to particles synthesized using this method.

### 2.2. Nanoparticles Synthesis: Laser Electrodispersion Method

The second synthesis method we employed for nanoparticle generation was LED, which is based on the ablation of a metallic target under the influence of a high-power pulsed periodic laser [22]. The synthesis of nanoparticles via laser ablation requires harsh irradiation regimes and conditions under which the ejected microdroplets of the target metal are efficiently fragmented into nanoparticles. This fragmentation becomes possible due to the high temperature of the laser plume plasma. In the plasma with an electron temperature of 20–30 eV, liquid metal droplets become charged up to a critical magnitude at which they turn unstable and begin to divide [23]. This droplet division process, arising due to capillary instability, has been studied in sufficient detail [24]. It results from the dominance of Coulomb repulsion forces over surface tension forces. Upon reaching the instability threshold, the droplet loses its spherical shape and begins to divide, generating a multitude of smaller daughter droplets [25]. This division is cascade-like, and the droplet size decreases by approximately an order of magnitude at each cascade stage. At the same time, the electric field at their surface increases, leading to an enhanced field emission (autoemission) of electrons. When the droplet size reaches a few nanometers, the flux of electrons emitted from the surface exceeds the flux of electrons arriving from the plasma; consequently, the droplets become stable, and their further division ceases [23]. Thus, the division of micron- and submicron-sized droplets in the laser plume plasma results in the formation of a vast quantity of nanoparticles and a small amount of residual mother droplets that failed to fully divide. The size of the nanoparticles, given the specified electrophysical parameters of the system, is a function solely of the metal’s nature [23,24].

In this study, a gold target (14 mm in diameter and 2 mm thick) was irradiated using a Nd:YAG laser (Jupiter 1, Integrated Technologies, Sosnovy Bor, Russia) with a wavelength of 1.06 µm, a pulse duration of 25 ns, and a pulse energy of 220 mJ. The laser beam was focused onto the target surface into a 1 mm diameter spot, resulting in a power density of approximately 10^9^ W/cm^2^ on the target surface. The LED deposition was performed in a 56 L vacuum chamber, which houses the target assembly and the substrate holder assembly in its center. The chamber was evacuated to 10^−7^ mbar using a turbomolecular pump (TSU 071, Pfeiffer Vacuum, Asslar, Germany). The distance between the target and the substrate was 60 mm, which allowed for optimal focusing of the laser radiation onto the target surface. HOPG plates were also used as substrates. The nanoparticles were deposited onto a substrate at 293 K.

The structure of nanoparticles synthesized using this method has been thoroughly investigated [22,26,27]. Results obtained via TEM and LEED indicate an amorphous structure for the metal particles [26], meaning that the variance in interatomic distances and angles differs significantly from that in a single crystal. The high corrosion resistance of such nanoparticles indirectly confirms their amorphous structure as well [26]. Hereinafter, amorphous nanoparticles will refer to those synthesized using the LED method.

### 2.3. STM Characterization and Exposition Experiments

The morphology and surface electronic structure of the synthesized single nanoparticles were determined by means of scanning tunneling microscopy and spectroscopy (STM/STS). In the case of the nanocontact formed between a metal sample and a conducting tip, the dependence of the STM tunnel current on voltage (current-voltage characteristic, CVC) forms a smooth S-shaped curve [28,29]. Changes in the surface elemental composition, for example due to chemisorption, can lead to a significant decrease in the density of states near the Fermi level, resulting in the zero-current region on the S-shaped curve [28,29]. The extent of this region, accurate to within a dimensional prefactor, corresponds to the local band gap width of the investigated sample. Thus, the shape of the CVC indicates changes in the chemical composition of the nanoparticle surface. The high spatial resolution of this method (~0.1 Å) allows for monitoring local variations in electronic structure.

All measurements were conducted under ultra-high vacuum (UHV) conditions. This approach prevented uncontrolled changes in the sample’s chemical composition due to residual gases. The working element of the scanning tunneling microscope (UHV VT STM, Omicron NanoTechnology, Taunusstein, Germany) utilized tungsten probes, fabricated via the standard method of electrochemical etching and subsequently treated with argon-ion bombardment in UHV conditions. In the experiments, we only used those probes that demonstrated reproducible S-shaped CVC curves when scanning the HOPG surface. Each such curve represents an average of 50 current-voltage measurements of the tunnel junction, formed by the STM probe held stationary over a selected point on the sample surface. The operational bias voltage range was from −2 V to +2 V. The bias voltage error is ±0.1 mV, and the tunnel current measurement error is ±10 pA.

The composition of the gas environment within the system was monitored throughout all stages of the experiment using mass spectrometric measurements performed with a quadrupole mass spectrometer (HAL 301 PIC, Hiden Analytical Limited, Warrington, UK). In the experiments described below, the molecular hydrogen pressure did not exceed *P* = 1·10^−6^ Torr, and the duration of sample exposure to hydrogen was selected based on the required exposure dose. Exposure was measured in Langmuirs (1 L = 1·10^−6^ Torr·s). Following exposure, hydrogen was pumped out from the chamber, and the sample was maintained under UHV conditions for 15–20 h to allow for the relaxation of all dynamic processes induced by the interaction of nanoparticles with the gas. Subsequently, changes in the morphology and electronic structure of the nanoparticles were monitored using STM/STS.

## 3. Results and Discussion

### 3.1. Crystal Nanoparticles Before and After Exposure to H_2_

**Figure 1:**
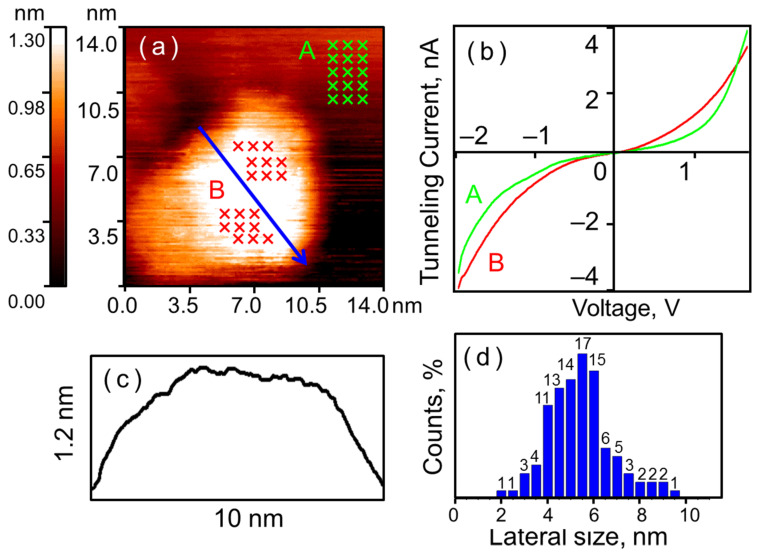
Crystalline gold nanoparticles on HOPG after annealing. STM/STS measurement results: (**a**) surface of HOPG with a single gold nanoparticle; (**b**) current-voltage characteristics of the tunnel nanocontact on the HOPG surface (green curve A) and on the nanoparticle (red curve B), averaged over the set of points marked with crosses in (**a**); (**c**) surface profile along the cut line indicated by the blue arrow in (**a**); (**d**) lateral size distribution of the investigated particles.

Following precursor decomposition, a coating of crystalline gold nanoparticles is formed on the HOPG surface (see Figure 1). The surface coverage is approximately 20% (counted manually). The nanoparticles exhibit a rounded shape. The maximum of the lateral size distribution lies in the 4–6 nm range, with an average height of 1.5–2 nm. The nanoparticles preferentially agglomerate at the edges of graphite terraces and other substrate surface defects; however, numerous individual nanoparticles are also observed. Spectroscopic measurements revealed that the CVCs of the nanoparticles and the graphite are similar, both exhibiting smooth S-shaped curves characteristic of a tunneling contact between two conductors. It is worth noting that the absolute value of the tunnel current measured on the nanoparticle is slightly higher than that measured on the graphite across the entire voltage range. This indicates that the local conductivity of the nanoparticles exceeds that of the graphite. This is expected, as the density of electronic states near the Fermi level is higher for metal nanoparticles than for HOPG [30].

**Figure 2:**
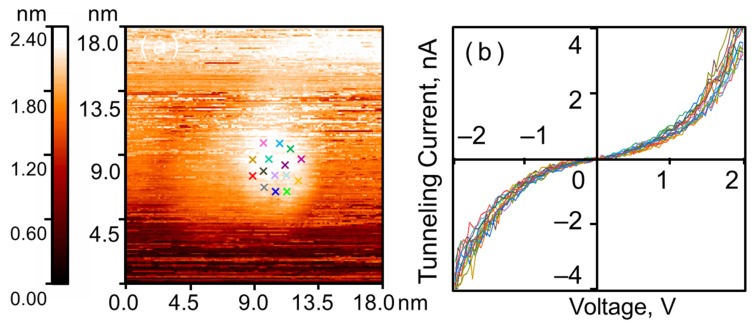
Tunnel current spread on the surface of a single crystalline nanoparticle. STM/STS measurement results: (**a**) surface of HOPG with a single gold nanoparticle; (**b**) CVCs of the tunnel junction, measured at a random set of points marked with colored crosses in (**a**) correspondingly.

The CVCs measured at various points on the nanoparticle surface exhibit some spread in current (see Figure 2), which, under otherwise equal conditions, indicates differences in the local density of states (LDOS). Calculating the LDOS from STM/STS measurement results is a separate, complex task, the solution of which subsequently allows for the estimation of surface chemical activity, for example, by calculating the binding energy with the adsorbate. However, as this task falls outside the scope of the present study, we will limit ourselves to a more general parameter that allows us to characterize the current variability and thereby indirectly describe the differences in LDOS across the nanoparticle surface.

Let’s randomly select *M* points on the surface of a single nanoparticle. For each such point, indexed by *j* = 1, 2… *M*, the CVC curve is represented by a set of *N* tunnel current values measured at bias voltages *U_i_*, where *i* = 1, 2… *N*. The CVC, averaged over the surface:

(6) $$I_{0}\left(U_{i}\right)=\frac{1}{M}\sum_{j=1}^{M}I_{j}\left(U_{i}\right)\;$$

As a measure of the deviation of each measured CVC from the averaged one, we introduce the following:

(7) $${\sigma_{j}}^{2}=\frac{1}{N}\sum_{i=1}^{N}{\left[I_{j}\left(U_{i}\right)-I_{0}\left(U_{i}\right)\right]}^{2}$$

while the general characteristic of the surface in this case is as follows:

(8) $$\bar{\sigma^{2}}=\frac{1}{M}\sum_{j=1}^{M}{\sigma_{j}}^{2}$$

This parameter is calculated from the CVC measurements for a random set of *M* points on the surface of a single crystalline nanoparticle. In our case, *M* = 15, and each CVC curve is represented by a set of *N* = 101 voltage-current pairs. For the CVCs shown in Figure 2, the obtained general measure of current spread was $\bar{\sigma^{2}}=0.04\;{nA}^{2}$.

**Figure 3:**
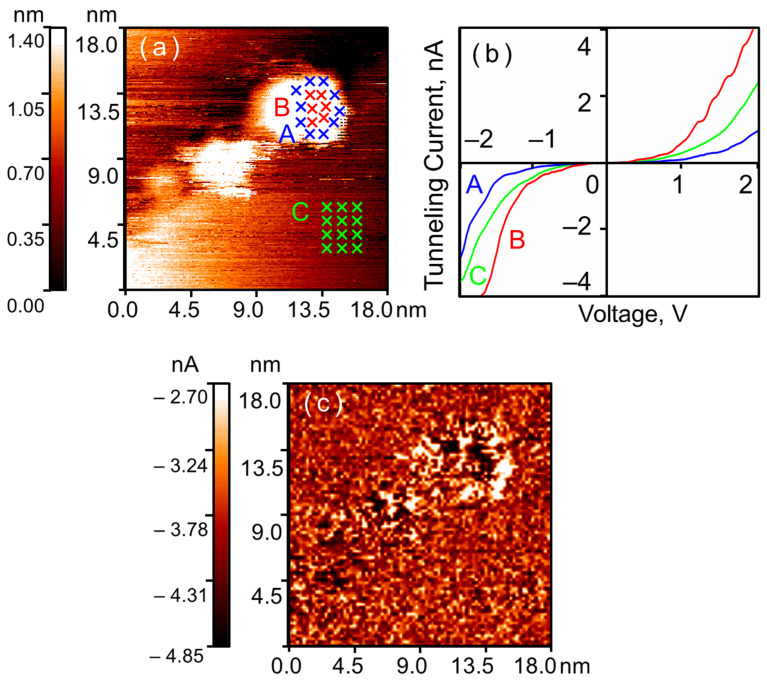
Agglomerates of crystalline gold nanoparticles after exposure to hydrogen (400 L, 10^−6^ Torr). STM/STS measurement results: (**a**) HOPG surface with gold nanoparticles; (**b**) averaged CVCs corresponding to the points indicated in the topography; (**c**) current image at *U* = –2.0 V. Blue curve A—nanoparticle surface near the Au–HOPG interface; red curve B—central region of the nanoparticle; green curve C—HOPG surface.

Exposure of the crystalline nanoparticles to 400 L of hydrogen significantly alters their electronic structure (see Figure 3). Along the outer edge of each nanoparticle, a region appears where the tunnel current reverses to zero within a certain range of bias voltages—a zero-current section is observed on the CVC curve. Furthermore, along the nanoparticle perimeter, a series of points is detected where the tunnel current is still measurable at the same bias voltages but is significantly lower than that on the HOPG. Meanwhile, the tunnel currents measured in the center of the nanoparticle still exceed the currents measured on the graphite. Thus, after exposure to hydrogen, two regions are observed on the surface of each crystalline gold nanoparticle: a central region with enhanced conductivity and a ring-shaped peripheral region with reduced conductivity. This result is observed even for nanoparticles within agglomerates and is in full agreement with our previously obtained results [13,14,15].

The connection between hydrogen adsorption on gold and the emergence of a zero-current region on the CVC is also supported by quantum chemical simulation results. For Au_13_H_12_, Au_55_H_39_, Au_147_H_86_ and other similar systems, it was demonstrated that hydrogen chemisorption leads to a reduction in the density of electronic states of gold near the Fermi level [31]. This effect was also shown to be local: a hydrogen adatom distorts the electronic structure of only the nearest gold atoms [31].

### 3.2. Amorphous Nanoparticles Before and After Exposure to H_2_

**Figure 4:**
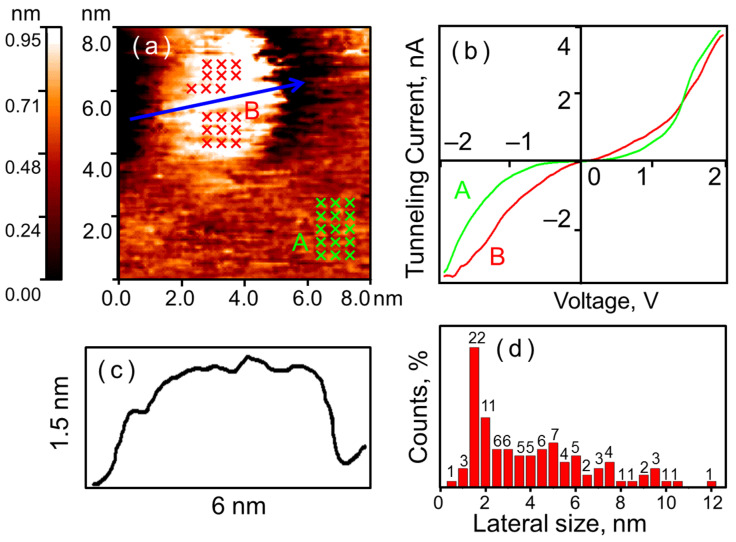
Amorphous gold nanoparticles on HOPG. STM/STS measurement results: (**a**) HOPG surface with a single gold nanoparticle; (**b**) CVCs of the tunnel nanocontact on the HOPG surface (green curve A) and on the nanoparticle (red curve B), averaged over the set of points marked with crosses in (**a**); (**c**) surface profile along the cut line indicated by the blue arrow in (**a**); (**d**) lateral size distribution of the investigated particles.

The LED synthesis method results in the formation of amorphous gold nanoparticles on the HOPG surface (see Figure 4). The surface coverage is approximately 10% (counted manually). Amorphous nanoparticles, like the crystalline ones, exhibit a rounded shape. The size distribution indicates that about 30% of the nanoparticles have a lateral size of 1.5–2.5 nm. Both single nanoparticles and agglomerates are observed on the HOPG surface.

**Figure 5:**
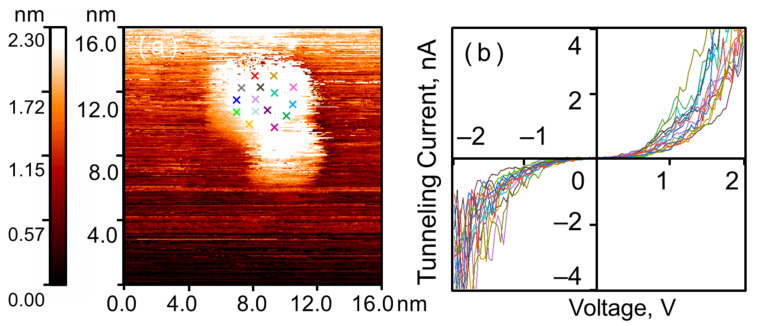
Tunnel current spread on the surface of a single amorphous nanoparticle. STM/STS measurement results: (**a**) HOPG surface with a single gold nanoparticle; (**b**) CVCs of the tunnel nanocontact, measured at a random set of points marked with colored crosses in (**a**) correspondingly.

As in the case of crystalline gold nanoparticles, the CVCs on the surface of amorphous ones correspond to a tunnel nanocontact formed by clean metals (see Figure 4b). As expected, the local tunnel currents measured on the nanoparticles exceed those measured on the HOPG surface. It is worth noting that the spread of tunnel currents within a single amorphous nanoparticle is noticeably larger than that of crystalline ones (see Figure 5). The same approach as in the previous case was applied to estimate the current spread. The general parameter was $\bar{\sigma^{2}}=0.29\;nA^{2}$, which is 7.25 times higher than that for crystalline nanoparticles. This indicates that the local electronic structure varies significantly across the surface of amorphous nanoparticles, and consequently, so does the probability of Au–H adsorption complex formation.

We should note that $\bar{\sigma^{2}}$ parameter is quite crude. For example, it does not account for the dependence on bias voltage, which is obvious from Figure 2b and Figure 5b. Strictly speaking, we need to use a minimal continuous positive function that, at any given voltage, exceeds the squared deviation of any current curve from the averaged one. However, such an elaboration is unwarranted within the scope of our work, as this parameter is introduced solely to provide a simple quantitative estimate of the observed fact—namely, that the current curves in Figure 2b cluster together, while they diverge in Figure 5b. For qualitative assessments, it is sufficient for us that this pattern is visually reproduced for all crystalline and amorphous nanoparticles we examined.

**Figure 6:**
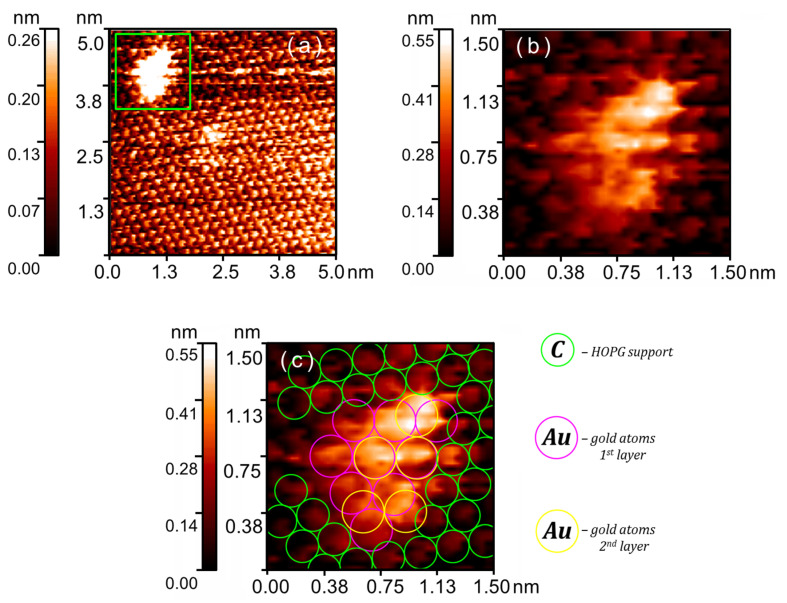
Gold LED nanoparticle with locally disordered atomic structure. STM/STS measurement results: (**a**) single nanoparticle on HOPG; (**b**) magnified image of the area highlighted by the green frame in (**a**); (**c**) reconstruction of the gold atomic arrangement in the selected nanoparticle. Pink and yellow circles represent gold atoms of the first and second nanoparticle layers, respectively, with *D*_Au_ = 0.292 nm; green circles represent elements of the triangular pattern of the top graphite layer with *D*_C_ = 0.229 nm.

Gold LED nanoparticles 1.5–2 nm in size are of particular interest (see Figure 6). Such nanoparticles are hardly formed when synthesized via the impregnation method. Direct reconstruction of the gold atoms packing confirmed the structure of the nanoparticles. The reconstruction of the ultra-small gold particle shown in Figure 6 reveals that its bottom layer consists of nine atoms and structurally corresponds to the Au(111) surface. This is presumably a consequence of the interaction with the substrate, where the hexagonal graphite lattice acts as a template for the distribution of gold atoms. Notably, the pattern orientations of HOPG and gold are misaligned by 12 ± 1°. The five atoms of the second layer in the nanoparticle are arranged more chaotically and show no evidence of periodical packing. Thus, even the smallest particles possess a structure with broken periodicity. The designation ‘amorphous’ is not strictly accurate for particles measuring 1.5–2 nm. From general principles, it can be said that such particles do not yield a distinct diffraction pattern in X-ray experiments. Nevertheless, they preserve vestiges of short-range order; we can see that the underlying layer of gold atoms follows the triangular pattern of graphite. Commonly, as a compromise, these particles are termed ‘X-ray amorphous,’ signifying the absence of long-range order within the system, without ruling out short-range order. Within the scope of this study, utilizing STM/STS rather than X-ray diffraction, we consider it more precise to describe their structure as ‘locally disordered atomic structure,’ which more faithfully represents the direct observation data obtained in our experiment. It should be noted that the term ‘Au(111) surface’ is also not strictly applicable to ultra-small nanoparticles, but is used by us due to the most accurate description of the triangular atomic packing pattern that we observe.

The observed gold atom size in the nanoparticle is *D*_Au_ = 0.292 nm. Bulk gold has a face-centered cubic (FCC) lattice with *a* = 4.0782 Å [32]. Consequently, the interatomic distance is *d*_Au_ = *a*/√2 = 2.8837 Å. The relative error in this case is *ε*_Au_ = (*D*_Au_ − *d*_Au_)/*d*_Au_ = 1.4%.

We use the HOPG lattice as a reference, which appears as a triangular pattern due to the nature of the electron density distribution within the graphite unit cell. Under scanning conditions, only three of the six atoms are observable [33,34]. The distance between them is *d*_C_ = 2.4612 Å [32]. Our experimental value is *D*_C_ = 0.229 nm, resulting in a relative error of *ε*_C_ = (*D*_C_ − *d*_C_)/*d*_C_ = 6.9%. Thus, the error in determining the interatomic distance for the gold nanoparticle doesn’t exceed the reference error.

**Figure 7:**
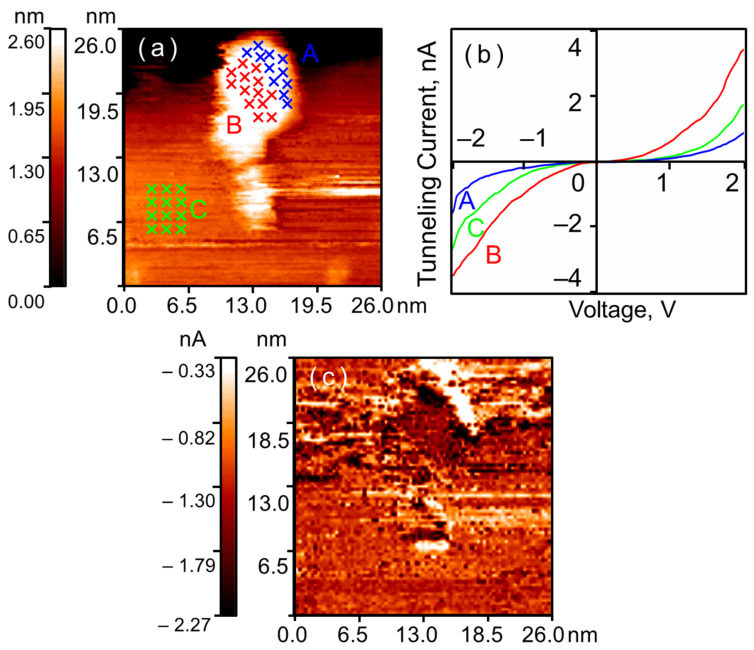
Amorphous gold nanoparticle after exposure to hydrogen (400 L, 10^−6^ Torr). STM/STS measurement results: (**a**) HOPG surface with a single gold nanoparticle; (**b**) averaged CVCs corresponding to the points indicated in the topography; (**c**) current image at *U* = –1.5 V. Blue curve A—nanoparticle surface near the Au–HOPG interface; red curve B—central region of the nanoparticle; green curve C—HOPG surface.

Exposure to 400 L of hydrogen locally alters the electronic structure of amorphous nanoparticles (see Figure 7). A region of reduced conductivity appears along their edge. At the center of the nanoparticle, the tunnel current remains higher than that measured on graphite. However, for amorphous nanoparticles, these regions of reduced conductivity appear as isolated spots near the visible edge. We practically never observe the formation of a continuous ring-like region of reduced conductivity.

The regions of reduced conductivity along the perimeter of amorphous nanoparticles exhibit a rounded shape. In our previous studies, we observed a ring-like region composed of three coalesced rounded areas on the surface of crystalline gold nanoparticles [14]. Apparently, regardless of the nanoparticle synthesis method, the growth of the hydride layer follows an island growth model. The formation of a continuous annular region is driven by the fact that the number of growth sites at the periphery of crystalline nanoparticles exceeds that in amorphous ones.

### 3.3. Size-Effect in Nanoparticle Hydrogenation

Increasing exposure to hydrogen for nanoparticles of both types leads to an expansion of the regions with reduced local conductivity. However, a significant difference in their growth rates is evident: full hydride layer coverage is achieved at substantially different exposure levels for amorphous and crystalline nanoparticles. For crystalline nanoparticles, an exposure of 2000 L is sufficient; in this case, CVCs at most points on the nanoparticle surface exhibit a zero-current region, which is in full agreement with our previous results [13,14]. In contrast, amorphous nanoparticles demonstrate electronic structure stability upon hydrogenation. Even at an exposure of 6000 L, individual points and even entire regions exhibiting metallic conductivity—where CVCs retain a smooth S-shape—are still observable on their surface. This fact is in good agreement with experimental data on the catalytic properties of nanoparticles of this size [35]: catalytic processes proceed effectively when the binding energy between the reaction product and the catalyst is not excessively strong. We can conclude that, for amorphous gold nanoparticles, it is precisely their atomic structural inhomogeneity that promotes the formation of weakly bound adsorbed hydrogen species. This weakly bound hydrogen can actively participate in catalytic processes, and its formation is indirectly evidenced in STM/STS experiments by a decrease in the amount of strongly bound hydrogen.

This stability is particularly pronounced in the case of ultra-small LED nanoparticles with locally disordered atomic structure. Experiments revealed a complete absence of hydrogen chemisorption on nanoparticles smaller than 2 nm. Even at high exposures to hydrogen, they maintain their metallic electronic structure.

All these observations allow us to estimate the lower size threshold at which the interaction of hydrogen with amorphous nanoparticles becomes observable under experimental conditions. For crystalline nanoparticles, the situation is reversed: in our previous works, we determined an upper size threshold of 10 nm [36].

**Figure 8:**
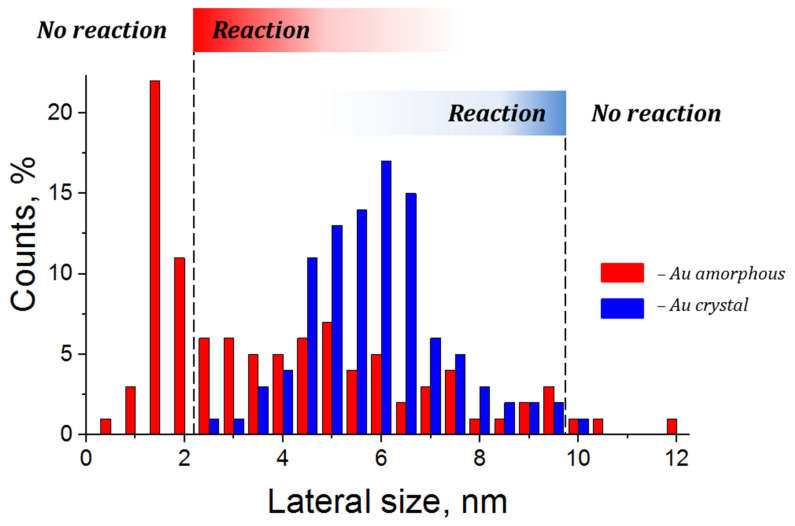
Lateral size distribution of amorphous and crystalline nanoparticles and the boundaries of the ‘optimal size window’ within which interaction with hydrogen leads to the formation of a hydride layer.

The existence of an ‘optimal size window’—and, consequently, two size thresholds between which the reaction becomes observable—is characteristic of chemically active nanosystems [37,38]. To accurately determine these thresholds, the nanoparticle size distribution must be broad enough to span this ‘window’. Unfortunately, this condition is not met in our case: the maximum size of the amorphous nanoparticles hardly exceeds 10 nm, while the number of crystalline nanoparticles smaller than 2 nm is insufficient for a reliable analysis (see Figure 8). The boundaries of this optimal size window may shift depending on the synthesis method, and additional studies are required to determine them.

It should be noted that in our case, the aspect ratio—the ratio of vertical to lateral dimensions—is approximately *f* = ⅓–½ for both amorphous and crystalline nanoparticles. On the one hand, this facilitates a valid comparison between the two types of nanoparticles. On the other hand, it does not allow us to determine which specific spatial parameter is responsible for the size effect in the studied systems. Consequently, there is significant interest in systems with *f* → 1 and *f* ≪ 1; however, in such cases, we are even more constrained by the limitations of existing synthesis methods.

## 4. Conclusions

The analysis of STM/STS results for amorphous and crystalline nanoparticles reveals important features of hydrogen chemisorption on their surface. Initially, both nanosystems exhibit a metallic electronic structure. For single crystalline nanoparticles, the conductivity of the tunnel nanocontact is nearly uniform across different surface points. Conversely, due to their inhomogeneous atomic structure, amorphous nanoparticles show a significant spread in the magnitude of the tunnel currents; this points to substantial differences in the local density of electronic states on the surface of the LED-synthesized nanoparticles, and consequently, to variations in their chemical activity.

The interaction of amorphous and crystalline nanoparticles with hydrogen leads to the formation of a surface gold hydride layer. For both nanosystems, the growth of the hydride layer begins at the periphery and follows an island growth model. At the periphery of crystalline nanoparticles, the number of growth sites is noticeably greater than in amorphous ones. With increasing hydrogen exposure, these surface hydride islands expand. On the surface of crystalline nanoparticles, the edges of the islands coalesce, eventually forming a continuous ring-like region along the nanoparticle perimeter. On the surface of amorphous nanoparticles, by contrast, a single hydride island predominantly expands, gradually covering the entire surface.

Despite the overall similarity in hydride layer growth, noticeable quantitative differences in the processes exist between the two nanosystems. Amorphous nanoparticles exhibit resistance to hydrogenation; surface coverage requires significantly higher hydrogen exposures (>6000 L) compared to crystalline nanoparticles (~2000 L).

In the case of ultra-small LED nanoparticles with locally disordered atomic structure, this stability is particularly pronounced. Even at high exposures to hydrogen, there are no signs of the hydride layer formation on their surface; thus, a size effect manifests itself. The lower size threshold in this case is 2 nm. To date, only the upper size threshold (10 nm) has been known for hydrogen chemisorption on crystalline gold nanoparticles [36]. Unfortunately, the second size threshold for each nanosystem remains unknown, and we cannot strictly determine how the boundaries of the ‘optimal size window’ for the gold hydrogenation reaction shift depending on the atomic structure. This issue can be addressed by collecting more statistical data. Such a study would require significantly more time, as particles of the necessary size are rarely encountered using the synthesis methods employed here.

Thus, STM/STS studies of gold nanoparticles have revealed a correlation between their atomic structure, local density of electronic states, and specific features of hydrogen chemisorption on their surface. This points to the possibility of introducing a local electronic descriptor that accounts for atomic packing and surface features. Most importantly, this descriptor can be determined experimentally via STM/STS! For instance, the analysis of CVCs allows, under certain assumptions, for the estimation of the surface DOS peak width and position relative to the Fermi level [39]. As previously noted, the contribution of surface electronic states to adsorbate binding in nanosystems can significantly exceed that of *d*-states [40]. The parameters of these specific states—their observation in the electronic structure of gold is described in [41,42]—could serve as a local electronic descriptor best suited for the analysis of heterogeneous reactions.

## Data Availability

The data presented in this study are available upon request from the corresponding author.
